# Triggering of Toll-like Receptor-2 in Mouse Myelomonocytic Leukaemia Cells WEHI-3B Leads to the Suppression of Apoptosis and Promotes Tumor Progression in Vivo 

**Published:** 2011

**Authors:** D.V. Shcheblyakov, D.Y. Logunov, I.V. Rakovskaya, M.M. Shmarov, B.S. Naroditsky, A.L. Ginzburg

**Affiliations:** Gamaleya Research Institute of Epidemiology and Microbiology, Russian Academy of Medical Sciences

**Keywords:** Toll-like receptor 2, synthetic diacylated lipopeptide Pam2CSK4, mouse myelomonocytic leukaemia cells WEHI-3B, transcription factor NF-kB, apoptosis; tumor progression

## Abstract

Toll-like receptors are the essential components of innate immunity. It is shown that TLRs play an essential role in the immune resistance of an organism to bacterial and viral infections. The binding of TLR to its own ligands results in the activation of several adapter molecules and kinases, inducing the activation of the main pro-inflammatory transcriptional factors, which in turn induce the activation of the main pro-inflammatory transcriptional factors. This activation results in the development of both the innate immune response triggered by the enhanced expression of a number of pro-inflammatory cytokines and antimicrobial peptides and that of the adaptive immune response, via the activation of dendritic cells and enhancement of antigen presentation, etc. The ability of TLR agonists to bolster the immune reaction makes them promising for use in the therapy of infectious diseases and in the chemotherapy of malignant neoformations. However, different TLR ligands may have either antitumor activity (lipopolysaccharide, imiquimod, CpG) or, conversely, could beef up the resistance of tumor cells to apoptosis, stimulating their proliferation under certain conditions (lipopolysaccharide, lipopeptide). It has been shown that the TLR2-dependent signalling pathway in the myelomonocytic mouse leukaemia cell line WEHI-3B leads to the constitutive activation of the transcriptional factor NF-kB, suppression of apoptosis in tumor cells, and progression of myelomonocytic mouse leukaemia*in vivo*, upon the addition of TLR2 agonist (synthetic lipopeptide Pam2CSK4) or following the infection of tumor cells with*Mycoplasma arginini. *

## INTRODUCTION

It is known that toll-like receptors (TLRs) are the crucial components of innate immunity and that they participate in the recognition of conserved pathogen-associated molecular patterns (PAMPs) and damage-associated molecular patterns (DAMPs) [1, 2]. The interaction between bacterial structures or DAMPs and specific Toll-like receptors initiates the development of reactions of both the innate and adaptive immune responses to induce the elimination of the causative agent from the organism [3, 4].

At the moment, thirteen human Toll-like receptors (TLR–13) are known. The majority of them are located on the surface of various immune cells (macrophagues, dendritic cells and mast cells, neutrophils, B-cells and T-cells, natural killer cells) and on nonimmune cells, such as fibroblasts, epithelial cells, keratinocytes, etc. [5–7]. The interaction between TLR and specific ligands initiates a cascade of signals originating from the cytoplasmic TIR domains of TLR [8]. The signal proceeds from the TIR domain through the adaptor molecules MyD88 (myeloid differentiation factor 88), TIRAP (TIR-domain-containing adaptors), TICAM1 (TRIF), TICAM2 (TIR-containing adaptor molecule) to the corresponding kinases (TAK, IKK, TBK, MAPK, JNKs, p38, ER K, Akt, etc.), providing differential activation of the transcription factors (NF-kB, AP-1, and IRF) that are responsible for the expression of various pro-inflammatory and antimicrobial factors (IL-6, IL-8, TNF, IL-1β), as well as for the activation of antigen-presenting cells [7, 9].

TLR have been shown to play a key role in the regulation of the adaptive immune response. Thus, the TLR-dependent activation of antigen-presenting dendritic cells is a crucial moment in several processes that are essential for the development of adaptive immunity, such as for the activation of mature T-cells; for the processing and presentation of microbial antigens; for boosting the expression of costimulatory molecules (СD80, CD86), which is required for the activation of naive CD4 ^+^ -T-cells; and for the suppression of regulatory T-cells via IL-6 production [8, 10]. It was also discovered that TLR-dependent activation is essential for B-cell maturation during the infection process [11]. Thus, TLR play a significant role in the organism, a role that consists in the development of inflammatory reactions (activation of the innate immune system) in response to various pathogens (protozoa, fungi, bacteria, and viruses) entering the organism [12]. 

The impact the expression and activation of Toll-like receptors has on tumor progression is currently a subject of wide-ranging discussion. It has been demonstrated that TLR can have a dual effect on tumor cells, depending on the following factors: TLRs or their ligands type, tumor type, administration method, and ligand concentration [13]. On the one hand it has been shown that TLR can activate an anti-tumor immune response [14, 15]. Numerous TLR agonists are currently in clinical trials for prospective application as anti-tumor agents. Thus, the natural (ssRNA) and synthetic (imiquimod) TLR7 and TLR8 agonists have a demonstrable high activity with respect to chronic lymphocytic leukaemia and skin cancer [16]. The TLR9 – CpG ligand is capable of suppressing the development of lymphomas, as well as brain, kidney, and skin cancer [14]. The TLR3 – poly(IC) ligand possesses proapoptotic activity not only against tumor cells, but also against the cells surrounding the tumor (e.g., endothelial cells).

However, despite the existing data on the anti-tumor activity of TLR agonists, numerous studies have recently been published that demonstrate that TLR ligands can enhance the progression of different types of tumors [15–17]. The TLR level is known to be high in various tumor cells; the frequency of induced tumor formation is decreased in TLR-knockout mice [18]. Furthermore, the boost in TLR expression on the cell surface of prostate tumors or head and neck tumors lead to an increase of the proliferation rate of these cells [19, 20]. Huang  *et al.* [20] demonstrated that *Listeria monocytogenes * possesses a direct tumor-stimulating effect associated with its ability to activate TLR2-dependent signal pathways in ovarian cancer cells. In addition, the TLR2-dependent activation of NF-kB induced by *L. monocytogenes* has been shown to increase the resistance of tumor cells to the action of chemotherapeutic agents [16]. The relationship between TLR2 and tumor progression was confirmed by Karin *et al.* [21], who proved that this receptor plays the key role in the metastasis of lung cancer. 

Thus, the dual effect of TLR indicates that its functional role in tumor biology is most complex, and that it requires a systematic investigation based on various models. 

An analysis of the TLR2 expression in various tumor cell lines was carried out in our laboratory. It was shown, using the model of the myelomonocytic mouse leukaemia cell line WEHI-3B, that the activation of the TLR2-dependent signalling pathway leads to apoptosis suppression and enhanced tumor growth *in vivo* , following the enjection of synthetic diacylated lipopeptide Pam2CSK4. A similar effect was observed for WEHI-3B cells infected with *Mycoplasma arginini* . It was revealed that micoplasma infection or the addition of the TLR2 agonist – diacylated lipopeptide Pam2CSK4 – to WEHI-3B cells results in the TLR2-dependent activation of the NF-kB transcription factor in tumor cells and the suppression of apoptosis induced by the action of various anti-tumor agents. Moreover, it was demonstrated on a model of myelomonocytic mouse leukaemia *in vivo * that the intramuscular introduction of Pam2CSK4 results in greater tumor resistance to the action of 5-fluorouracil, enhancement of tumor growth, and a reduction in the survival rate of mice. An analysis of the mechanism of the previously described effect of the TLR2 agonist on WEHI-3B cells demonstrated that the activation of the NF-kB factor, as well as the stimulation of the secretion of a number of pro-inflammatory cytokines (which are growth and development factors of myelomonocytic tumors), plays the key role in faster tumor progression. 

## EXPERIMENTAL

**Cell lines**

 TLR2 expression was analyzed in the myelomonocytic mouse leukaemia cell line WEHI-3B, transformed murine macrophages В10М, murine fibroblasts L929, human leukaemic monocyte lymphoma U937 cells, human lung cancer cellsA549 and H460, human nonsmall cell lung cancer H1299 cells, human large intestine cancer HCT116 cells, and human breast cancer MCF-7 cells. The following were analyzed in WEHI-3B cells: the activity of NF-kB, caspases-3/7, viability, the mitochondrial transmembrane potential, and the proliferation rate. 

WEHI-3B cells were cultured in a RPMI medium with 10 vol % of fetal bovine serum (catalogue number SV30160.03, Hyclone, USA), 1 mg/ml glutamine (catalogue number F032, PanEco, Russia), 50 U/ml penicillin, and 50 µg/ml streptomycin (catalogue number A065, PanEco, Russia) at 37°С in 5% CO _2_ . Cells were seeded at a 1 : 6 ratio on day 2. 

**Bacterial strains**

 The micoplasma strain * Mycoplasma arginini * used in this study was kindly provided by I.V. Rakovskaya (Laboratory of Mycoplasmas and Bacterial L-Forms, Gamaleya Research Institute of Epidemiology and Microbiology, Russian Academy of Medical Sciences). A flow cytometry kit (Bender Medsystems FlowCytomix, Austria) was used to determine the concentrations of chemokine and cytokine. 

**Reverse transcription reaction**

**Fig. 1:**
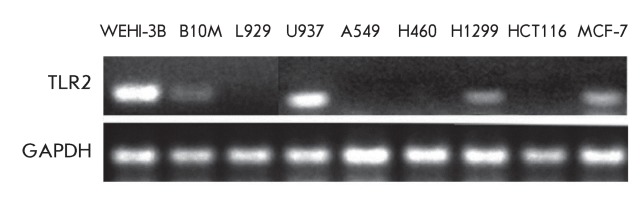
Analysis of TLR2 expression in different tumor cell lines.

 The expression of *TLR2 * genes in different human/murine cell lines was determined by RT-PCR. The total RNA was isolated using TRIZOL reagent (Invitrogen). The reverse transcription reaction was performed using an RT System kit (Promega). cDNA of the human/mouse *TLR2* and *GAPDH* genes were PCR-amplified using the following primers: mouse *TLR2* gene – upstream primer 5’-gtttccttctgaccaggatc-3’, downstream primer 5’-gcagcatcattgttctcttc-3’; human *TLR2* gene – upstream primer 5’-acctgtgtgactctccatcc-3’, downstream primer 5’-gcagcatcattgttctcttc-3’; human *GAPDH* gene – upstream primer 5’-tctagacggcaggtcaggtccacc-3’, downstream primer 5’-ccacccatggcaaattccatggca-3’; mouse *GAPDH* gene – upstream primer 5’-gcatcttcttgtgcagtgcc-3’, downstream primer 5’-tcacacccatcacaaacatg-3’. 

**Measurement of β-galactosidase activity **

 The culture medium was removed 24 h after the specimens had been added to the cells, and a lysis solution with β-galactosidase substrate (1 mM MgCl _2_ ; 0.25 M Tris-HCl pH 7.4; 0.02% NP40; 2 g/l *o* -nitrophenyl-β- *D* -galactopyranoside (catalogue number 102473, MP Biomedicals, USA) was then added. β-galactosidase activity was determined spectrophotometrically (414 nm) based on the conversion of the substrate ( *o* -nitrophenyl-β- *D* -galactopyranoside) into the colored product *o* -nitrophenol. 

**Cell viability analysis**

 Cell survival was assessed based on the ratio (%) between the intensity of the cells stained with methylene blue (pre-treated with cisplatin, taxol, and fluorouracil at varying concentrations) and the control, untreated cells (methylene blue was extracted with 0.1% SDS, its amount was determined chromatographically). 

**Caspases-3/7 activity measurement**

 The measurement of caspase activity was performed with the use of a specific to caspase-3/7 fluorogenic substrate Ac-DEVD-AMC (30 µM in lysis buffer pH 7.0 containing 10 mM HEPES, 0.4 mM EDTA, 0.1% CHAPS, 2% glycerol, and 2 mM DTT). Cells were incubated for 16 h with apoptosis-inducing drugs. The fluorescence intensity was measured immediately (after 0 h) and 6h after additions of a substrate. The measurement was performed using a Wallac 1420 plate reader (Perkin Elmer). 

**Measurement of the level of mitochondrial transmembrane potential (Δψ _m_ ) **

 The level of the mitochondrial transmembrane potential (Δψ _m_ ) was assessed on the basis of the binding of the fluorogenic dye DioC6 (Sigma, USA), of which the degree of specific binding with mitochondrial membranes is dependent upon the Δψ _m _ value. The cells infected with *M. arginini * were placed into a 24-well plate (10 ^5^ cells per well). DioC6 at a concentration of 40 nM was added to the cells following apoptosis induction. The cells were incubated for 30 min at +37 ^0^ С and washed twice with a phosphate buffer. The fluorescence was then measured using a Wallac 1420 plate reader. 

**Measurement of cytokine activity**

 BALB/c mice received 5 µg of Pam2CSK4 intramuscularly. Blood samples were collected, and the concentrations of 14 chemokines and cytokines (IL-1, -2, -4, -5, -6, -10 and -12, TNFα, MCP-1 and -3, MIP-1a, -1b, RANTES, interferon-γ) were determined in serum by flow cytometry using a FlowCytomix BenderMedsystems kit (Austria). 

**Laboratory animals**

 Six-week-old (by the beginning of the experiment) female BALB/c mice and D2&I thymus-free mice were used for this study. 

**Analysis of the survival rate of BALB/c mice**

 In order to assess the effect of diacylated lipopeptide Pam2CSK4 on the rate of tumor progression, WEHI-3B cells (2 × 10 ^6 ^ cells per mouse) were introduced intraperitoneally to BALB/c mice, which had been divided into different groups. 5 µg of Pam2CSK4 was introduced into each mouse at intervals of 1, 3, and 5 days post tumor transplantation. After 20 days, the mice were euthanized with diethyl ether; the liver and spleen were removed so as to be used for the macroscopic and histological analysis. In addition, the average weight of the spleen for each experimental group was determined. 

**Fig. 2:**
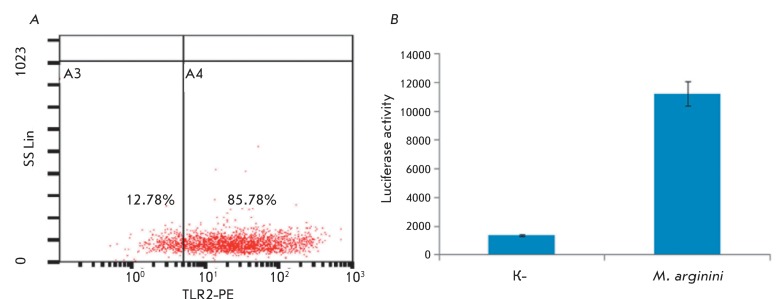
Activation of NF-kB in WEHI-3B cells in response to mycoplasmal infection or after treatment with diacylated lipopeptide Pam2CSK4. (А) – TLR2 expression in WEHI-3B cells. Expression of the specific for diacylated lipopeptides receptor TLR2 was additionally confirmed by flow cytometry. WEHI-3B cells were incubated with antibodies specific to mouse TLR2 (eBioscience, USA). Control cells were incubated with isotype control antibody (anti-IgG). The percentage of TLR2 positive cells was determined by phycoerythrin fluorescence (the assay was conducted according to the manufacture manual). (B) – NF-kB-dependent expression of luciferase gene. Luciferase gene under NF-kB – responsive promoter was introduced in WEHI-3B cells by lentiviral infection. NF-kB-dependent luciferase expression was measured using the standard procedure after *M. arginini* infection or treating the cells with diacylated lipopeptide Pam2CSK4.

The effect of the joint introduction of Pam2CSK4 and tumor cells on the animals survival rate was studied on BALB/c mice, to which WEHI-3B cells (2 × 10 ^6^  cells per mouse) were intraperitoneally transplanted. Synthetic diacylated lipopeptide (Pam2CSK4) was systemically introduced at a dose of 5 µg/mouse after 24 h. After 2 days, the mice also received Pam2CSK4 for a period of 3 days; the mice from the groups subjected to chemotherapy additionally received 0.6 mg of 5-fluorouracil. The control groups consisted of the animals that had received Pam2CSK4 and 5-fluorouracil only. Each group consisted of 10 mice. The animals were monitored until the death of the final mouse (32 days); the general condition of the mice was recorded. 

## RESULTS AND DISCUSSION

**Analysis of the expression of the Toll-like receptors 2 and 6 in different tumor cell lines**

 The model we used to study the effect of TLR2 on the proliferation of tumor cells and on tumor progression *in vivo* was selected via an analysis of the expression of the Toll-like receptor 2 in different tumor cell lines (WEHI-3B, B10M, L929, U937, A549, H460, H1299, HCT116, and MCF-7) by reverse transcription followed by PCR against the *TLR2 * gene * (Fig. 1).*

The Toll-like receptor 2 was expressed in five out of the nine cell lines analysed (WEHI-3B, B10M, U937, H1299 and MCF-7). The highest level of expression of this receptor was observed in the myelomonocytic mouse leukaemia cell line (WEHI-3B), which was subsequently selected for use as a model for the *in vitro* and *in vivo* experiments. 

**TLR2 agonist activates NF-kB and suppresses apoptosis in tumor cells WEHI-3B expressing Toll-like receptor 2**

**Fig. 3:**
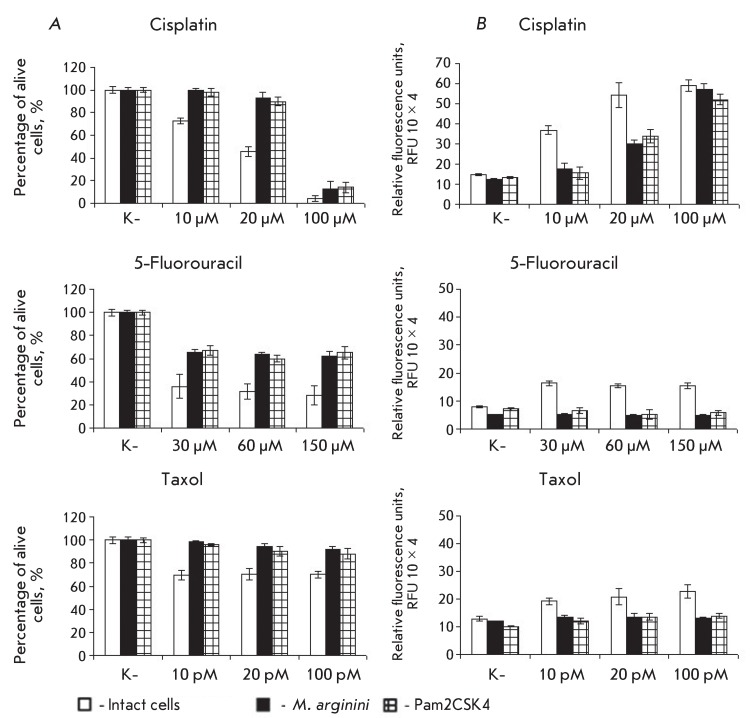
Survival and caspases 3/7 activity in myelomonocytic leukaemia cells WEHI-3B following exposure to chemotherapeutic agents at different concentrations. (А) – survival of myelomonocytic leukaemia cells WEHI-3B; (B) – caspases 3/7 activity. The data on each point is a result of three independent experiments. ( *р*  < 0.005).

**Fig. 4:**
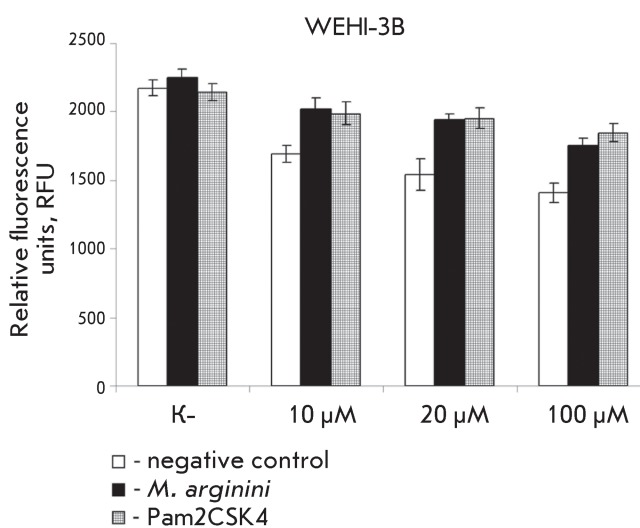
Membrane mitochondrial potential level (Δψ _m_ ) in WEHI-3B cells after exposure to different concentrations of cisplatin. The data on each point is a result of three independent experiments. ( *р*  < 0.005).

 Such parameters as the NF-kB factor activity ( *Fig. 2* ), cell survival rate, caspases-3/7 level ( *Fig. 3* ), and the level of the mitochondrial transmembrane potential (Δψ _m_ ) ( *Fig. 4* ) were measured at the next stage of the assessment of the effect of TLR2 agonists on apoptosis induced by chemotherapy drugs in WEHI-3B cells expressing the Toll-like receptor 2. WEHI-3B cells were infected with *M. arginini, * or the TLR2 agonist (synthetic diacylated lipopeptide Pam2CSK4) was added followed by treatment with cisplatin, taxol or fluorouracil. The parameters mentioned above were measured after 16–18 h of incubation. 

It was demonstrated that micoplasma infection of the tumor cells WEHI-3B containing the Toll-like receptor 2 resulted in the activation of the transcription factor NF-kB in these cells ( *Fig. 2* ). Similar results were obtained when synthetic diacylated lipopeptide was added to Pam2CSK4. 

*Fig. 2A* shows the data obtained by flow cytometry using specific antibodies to TLR2, which confirm the expression of TLR2, the major receptor of micoplasmal diacylated lipopeptides, and the data on the activation of NF-kB in WEHI-3B cells via the TLR2-dependent pathway ( *Fig. 2B* ). 

*Fig. 3* shows the survival rate and activity level of the major effector caspases-3/7 upon induction of apoptosis by chemotherapeutical drugs in WEHI-3B cells infected with *M. arginini,* or upon the addition of Pam2CSK4 to these cells. The level of caspases-3/7 was measured spectrophotometrically, using the specific fluorogenic substrate Ac-DEVD-AMC. As follows from these data, mycoplasma infection or the addition of lipopeptide Pam2CSK4 results in a statistically significant ( *р*  < 0.005) increase in the survival rate of WEHI-3B cells and a 25–30% decrease in the level of caspase-3/7 activation upon various intracellular damages in comparison with noninfected cells (white bars). 

**Fig. 5:**
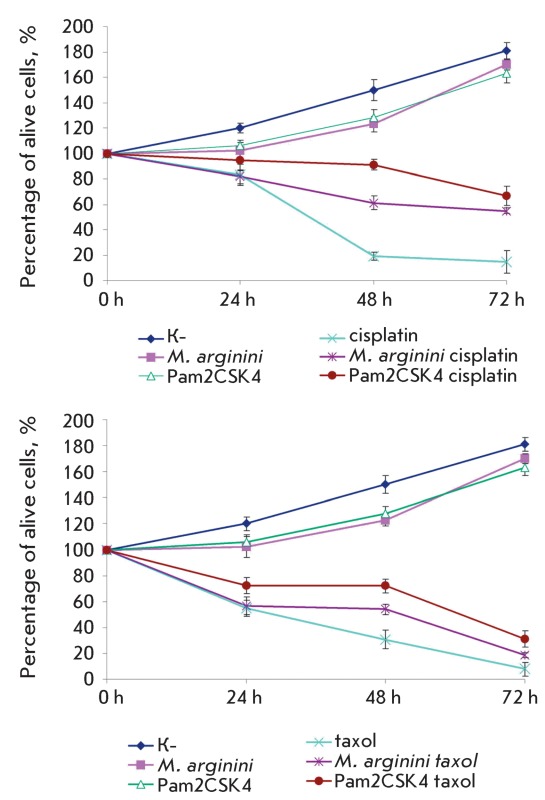
Proliferation rate of WEHI-3 cells. K – control cells; *M. arginini* – cells infected with *M. arginini* ; Pam2CSK4 – cells treated with diacylated lipopeptide; cisplatin – control cells treated with cisplatin; *M. arginini* cisplatin – cells infected with *M. arginini* and treated with cisplatin; Pam2CSK4 cisplatin – cells treated with diacylated lipopeptide and cisplatin; taxol – control cells treated with taxol; *M. arginini* taxol - cells infected with *M. arginini* and treated with taxol; Pam2CSK4 taxol – cells treated with diacylated lipopeptide and taxol. (р < 0.005).

The mitochondrial transmembrane potential (Δψ _m_ ) was also measured in WEHI-3B cells; a decrease in this potential caused by various stress factors is the major apoptotic marker. 

For this purpose, different concentrations of cisplatin were used to affect the infected or Pam2CSK4-treated cells; the level of the mitochondrial transmembrane potential (Δψ _m_ ) was measured after 16 h ( *Fig. 4* ). 

The level of Δψ _m _ was assessed based on the binding of the fluorogenic dye DioC6, the degree of specific binding to mitochondrial membranes, which is dependent upon Δψ _m_ . 

As can be seen from the data presented, the level of mitochondrial transmembrane potential in cisplatin-treated WEHI-3B cells infected with *M. аrginini* was higher by 25–30% than that of the noninfected cells (white bars), a point attesting to apoptosis suppression in the infected cells. Similar results were also obtained when using the synthetic diacylated peptide Pam2CSK4. 

Thus, it was demonstrated in the first path of the *in vitro* experiment that micoplasma infection or the addition of structural micoplasmal components to WEHI-3B cells expressing the Toll-like receptors 2 and 6 results in the activation of the transcription factor NF-kB in them and apoptosis suppression upon different types of intracellular damage. 

**Kinetics of the growth of tumor cells WEHI-3B upon infection with **

*M. arginini,* **or upon addition of diacylated lipopeptide Pam2CSK4 in the** *in vitro* **experiment**

The transcription factor NF-kB participates in the regulation of the expression of a number of proteins, including those controlling cell proliferation and apoptosis [19].

At the next stage of the study, we decided to observe the level of the impact that the micoplasmal infection or structural micoplasmal components, together with the anti-apoptotic activity, can have on both the kinetics and proliferation rates of the tumor cells WEHI-3B under normal conditions and/or upon the induction of apoptosis in them.

For this purpose, * M. arginini * or Pam2CSK4 was added to the WEHI-3B cell line selected as a model; the activation of Nf-kB in these cells was verified. The cells were then seeded into a 96-well plate (10 ^3^  cells per well), and apoptosis-inducing drugs (cisplatin and taxol) were added. The kinetics of cell growth was determined based on the accumulation of cell biomass (methylene blue staining) for 72 h (with or without the apoptosis stimulus). 

It follows from *Fig. 5* that apoptosis blockage was observed in micoplasma-infected cells with and/or upon addition of Pam2CSK4. However, no increase in the proliferation rate was observed. 

**Fig. 6:**
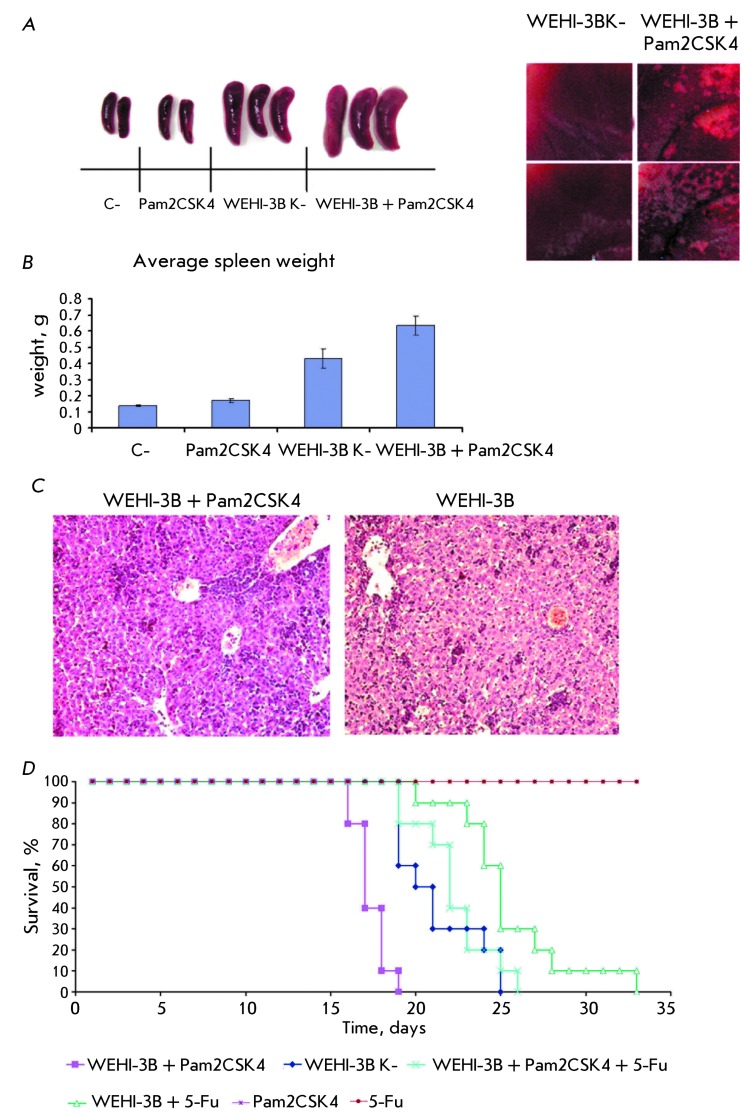
Influence of diacylated lipopeptide Pam2CSK4 on the proliferation rate and chemotherapy resistance of WEHI-3B tumor cells *in vivo* . (А) – macrophotographs of mouse organs. The macrophotograph of the spleen is presented on the left-hand side; the macrophotograph of liver sections with infiltrations is presented on the right-hand side. C – group of intact mice injected with PBS; Pam2CSK4– group of mice injected with diacylated lipopeptide Pam2CSK4; WEHI-3В – group of mice injected with WEHI-3B tumor cells; WEHI-3В+ Pam2CSK4– group of mice injected with WEHI-3B tumor cells and treated with Pam2CSK4. (B) – the average weight of spleens. Five organs from different groups of mice were used to determine the average weight. (C) – macrophotographs of a liver slice. Liver samples were placed into 10% formalin for fixation. The samples were then embedded into paraffin blocks according to the standard protocol; slides were stained with hematoxylin and eosin. (D) – survival curves of Balb/С mice. WEHI-3В K – group of mice with i.p. injected WEHI-3B cells; WEHI-3В + Pam2CSK4 – group of mice with i.p. injected WEHI-3B cells and i.m. injected Pam2CSK4; WEHI-3В + 5-Fu – group of mice with i.p. injected WEHI-3B and treated with 5-fluorouracil; WEHI-3В + Pam2CSK4 + 5-Fu – group of mice with i.p. injected WEHI-3B cells and i.m. injected Pam2CSK4 and treated with 5-fluorouracil ( *р* < 0.001).

**Fig. 7:**
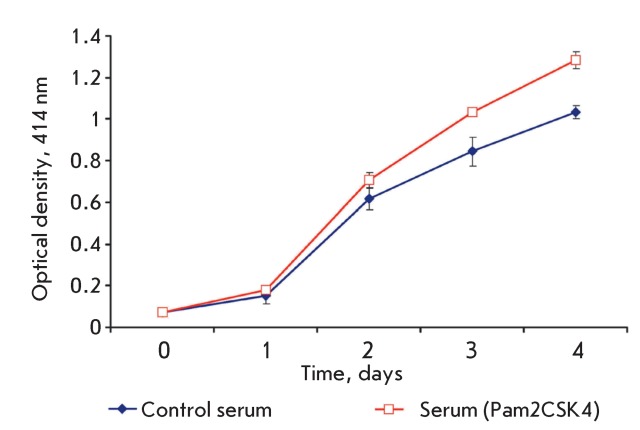
Proliferation rate of myelomonocytic leukaemia cells WEHI-3B after exposure to serum of mice injected with Pam2CSK4 (р < 0.005).

It was thus demonstrated that apoptosis suppression in micoplasma-infected cells caused by the activation of the transcription factor NF-kB does not result in an *in vitro* increase in the cell proliferation rate. 

**The effect of the TLR2 agonist – diacylated lipopeptide Pam2CSK4 – on the proliferation and resistance of WEHI-3B cells to chemotherapeutic agents in an **

*in vivo* **experiment**

The effect of the antigens circulating in the mouse’s organism (TLR2 agonists) on the proliferation and resistance of the myelomonocytic mouse leukaemia cells WEHI-3B to chemotherapeutic agents *in vivo * was studied. 

Firstly, the effect of the diacylated lipopeptide Pam2CSK4 on the rate of tumor progression was assessed. Forty 18–20 g animals (female BALB/C mice) participated in the experiment. The animals were divided into four groups, 10 mice per group. The control group (the first group) consisted of intact mice. The second group included mice that received three doses of Pam2CSK4 intramuscularly. The third group was comprised of the mice transplanted with WEHI-3B cells (2 × 10 ^6^ cells per mouse). The mice from the fourth group were intraperitoneally transplanted with WEHI-3B cells of identical dose. Each mouse from this group received 5 µg of Pam2CSK4 on days 1, 3, and 5 following tumor transplantation. The mice were euthanized with diethyl ether in order to assess the tumor progression after 20 days; the liver and spleen were removed from the mice and were subsequently used for macroscopic and histological studies. The average weight of the spleen was determined in each experimental group ( *Fig. 6A–B* ). According to the data obtained in the macroscopic study, no visible pathological changes were observed in mice from groups 1 and 2 ( *Fig. 6A* ). However, a negligible increase in the average weight of the spleen was observed in mice from group 2 ( *Fig. 6B* ). The changes typical of leukaemia (increase of spleen size and slightly swollen liver) were detected in the liver and spleen of the mice with transplanted myelomonocytic mouse leukaemia cells (group 3) by macroscopic studies. Sparse tumors were detected on the liver and spleen surface. When performing the macroscopic study of the liver and spleen obtained from mice transplanted with leukaemia cells and that had received Pam2CSK4 (group 4), the changes typical of leukaemia were also observed. The spleen was considerably swollen. Loose neo-formations (which turned out to be myelomonocytic leukaemia cells) were found on the spleen and liver surface. 

The measurements of the average weight of the spleen demonstrated a significant increase in the weight of this organ in mice with leukaemia (groups 3 and 4) as compared to the mice from the control groups 1 and 2. The introduction of Pam2CSK4 to mice from group 4 resulted in an even greater increase in the average weight of the spleen ( *p*  < 0.05) in comparison with the animals from group 3 ( *Fig. 6B* ). 

No pathological changes were revealed in a histological study of the spleen and liver of the mice from groups 1 and 2. An identical pattern was observed in the spleen of the mice from groups 3 and 4. Diffuse dense infiltration of pulp with myelomonocytic leukaemia elements was observed; lymphatic follicles were atrophied. The greatest differences were observed between the liver samples of the mice from groups 3 and 4. Numerous small myelomonocytic leukaemia infiltrates were present in the livers of the mice from group 3, whereas the mice livers from group 4 were considerably larger ( *Fig. 6C* ). Infiltrates mostly localized along sinusoids. Leukaemia cell aggregation was also detected in individual blood vessels. A clearly defined surface infiltration of a liver with leukaemia cells was observed in mice from group 4, in contrast to those from group 3. 

The macro- and microscopic studies of spleen and liver samples obtained from mice which were intraperitoneally transplanted with WEHI-3B cells allowed us to arrive at the conclusion that micoplasma diacylated lipopeptide promotes tumor progression.

**Fig. 8:**
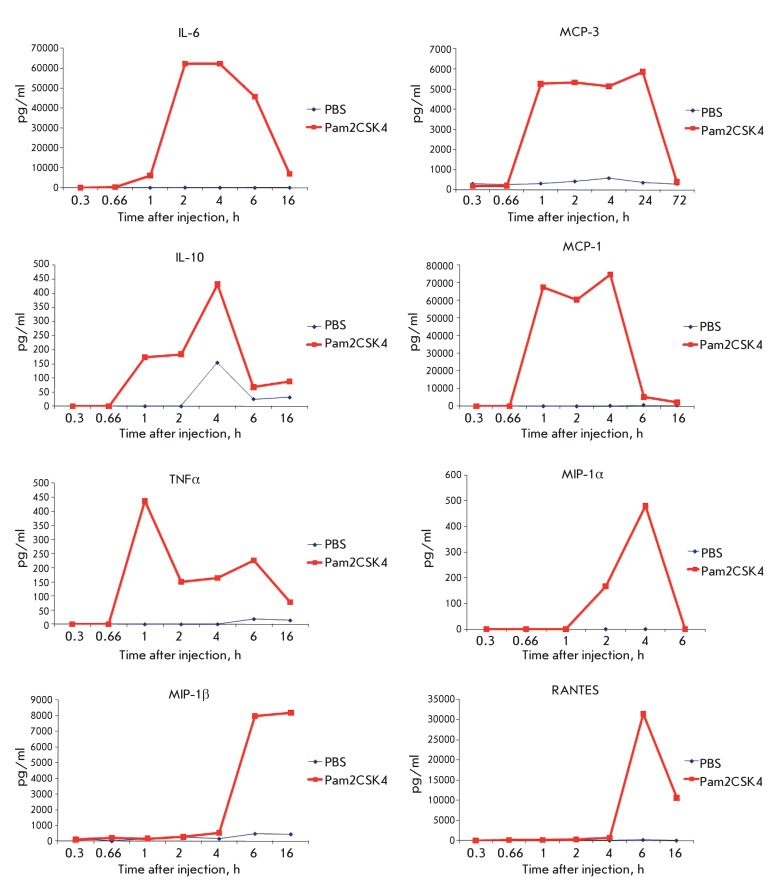
Determination of serum cytokine concentrations in mice injected with Pam2CSK4. BALB/C mice were injected with diacylated lipopeptide Pam2CSK4 or PBS. Blood samples were collected for serum preparation after the indicated time intervals. Cytokine concentrations were determined in serum samples. Each point is the average value of three independent experiments.

The effect of the diacylated lipopeptide Pam2CSK4 on the rate of tumor progression was assessed at the next stage. The nature of the effect of Pam2CSK4 on the resistance of the transplanted cells to chemotherapeutic agents was simultaneously determined. This experiment was performed according to the scheme described in the Experimental section. *Fig. 6D* shows the diagram of the survival rate of the experimental mice. 

It was shown via an analysis of the Kaplan–Meier survival curves that the mice receiving the synthetic diacylated lipopeptide demonstrated a less favorable response to 5-fluorouracil in comparison with those that did not receive Pam2CSK4. The last mouse from the group receiving chemotherapy died on day 33, whereas the mice that received Pam2CSK4 simultaneously with chemotherapy died as early as on day 26.

The *in vivo * survival rate of mice was in complete correlation with the results previously obtained on culture cells. Moreover, as follows from the diagram, all mice into which WEHI-3B and Pam2CSK4 cells were simultaneously introduced died as early as on day 19, whereas the lifetime of those mice that did not receive Pam2CSK4 was equal to 25 days. These data attest to the fact that the intramuscular administration of Pam2CSK4 results in accentuated tumor progression and a decrease in the lifetime of mice. It is noteworthy that these experimental results showed no agreement with the data obtained for a cell culture, where the addition of Pam2CSK4 into the culturе media did not result in an increase in the proliferation rate of WEHI-3B cells. 

**The effect of diacylated lipopeptide Pam2CSK4 on production of the factors stimulating the **

*in vivo* **proliferation of myelomonocytic mouse leukaemia cells WEHI-3B**

Taking into account the major difference between the *in vitro* and *in vivo * growth of WEHI-3B cells in the presence of Pam2CSK4, it was assumed that the factors that are essential for the proliferation of WEHI-3B cells can occur in the organism of the experimental animals after diacylated lipopeptide is introduced, thus determining the kinetics of the *in vivo* growth of these cells. 

To corroborate this hypothesis, we studied the effect of serum obtained from the mice that had received Pam2CSK4 on the proliferation rate of WEHI-3B cells. BALB/c mice received intramuscular injections of 5 µg of diacylated lipopeptide Pam2CSK4. Twenty-four hours after the injection, blood samples were taken to obtain serum. Serum from mice that received a phosphate saline buffer was used as a control. The sera were used to prepare 5% of the medium for culturing WEHI-3B (RPMI) cells. The media were added to WEHI-3B cells, which were then seeded into a 96-well plate at a concentration of 10 ^3 ^ cells per well. The kinetics of cell growth was determined based on the accumulation of cell biomass in the reaction with the MTT substrate during 72 h ( *Fig. 7* ). 

As follows from *Fig. 7* , the addition of blood serum obtained from the mice that intramuscularly received Pam2CSK4 to WEHI-3B cells resulted in an increase in their proliferation rate. The results of this experiment corroborated the assumption earlier made about the possible production of the factor inducing the growth of the myelomonocytic mouse leukaemia cells WEHI-3B in response to the introduction of Pam2CSK4. 

An attempt to identify the factors promoting the accentuated growth of WEHI-3B cells was undertaken at the next stage.

For this purpose, the synthesis of chemokines and cytokines in the organism in response to the introduction of diacylated lipopeptide was analyzed ( *Fig. 8* ). The cytokine expression was determined according to the procedure described in the Experimental section. *Fig. 8* shows the data for cytokines whose expression level changed in response to the introduction of Pam2CSK4. It is clear that the introduction of Pam2CSK4 resulted in a change in the expression of eight of the 14 cytokines. An analysis of the published data demonstrated that five of these cytokines (IL-6, MCP-1, MCP-3, RANTES, and TNFα) are capable of direct or indirect promotion of tumor growth [21]. 

Hence, it was shown that the activation of the TLR2-dependent signalling pathway in WEHI-3B cells after the introduction of diacylated lipopeptide Pam2CSK4 or *M. arginini * cells leads to the constitutive activation of the transcription factor NF-kB in WEHI-3B cells. In turn, the activation of NF-kB results in increased resistance of these cells to various assaults induced by chemotherapeutic agents (cispatin, taxol, and fluorouracil). 

It was demonstrated via *in vitro * experiments that apoptosis suppression in the cells infected with *M. arginini* , which was caused by the activation of the transcription factor NF-kB, had no effect on the rate of cell proliferation. However, the data obtained *in vivo* differs: the intramuscular introduction of Pam2CSK4 promoted the growth of myelomonocytic mouse leukaemia cells WEHI-3B in the organism of experimental animals. This fact is mostly accounted for by the ability of Pam2CSK4 to stimulate the expression of the factors (IL-6, MCP-1, MCP-3, RANTES, and TNFα) boosting the growth of tumor cells. 

The results obtained for the model WEHI-3B cells show that the activation of the Toll-like receptor 2 in tumor cells of myelomonocytic origin caused by mycoplasmal infection or the direct action of the TLR2 agonist (diacylated lipopeptide) promotes the growth of these cells. Meanwhile, studying the effect of mycoplasma and its structural component diacylated lipopeptide Pam2CSK4 on the development of the tumor allows one to arrive at the conclusion that the mycoplasmal infection may impact not only the rate of disease progression, but also the effectiveness of anti-tumor therapy. This observation is valid not only for mycoplasmas, but also for the other pathogens causing various infections in patients with malignancies. The potential exists to conduct efficient therapy in the case of myelomonocytic leukaemias, provided that the disease is not stimulated by the factors of pathogenic microorganisms, or their antigens, circulating in the body. 

## Abbreviations

TLR2: Toll-like receptor 2
NF-kB: nuclear transcription factor-kB
PAMP: pathogen-associated molecular pattern
DAMP: damage-associated molecular pattern
ssRNA: single*-*stranded**ribonucleic**acid
RT-PCR: reverse transcription polymerase chain reaction
TNF-α: tumor necrosis factor α
IL: interleukin
MCP1: monocyte chemoattractant protein 1
